# Healthcare costs of transarterial chemoembolization in the treatment of hepatocellular carcinoma

**DOI:** 10.2147/JHC.S144068

**Published:** 2017-10-16

**Authors:** Waleed Fateen, Farooq Khan, Richard J O’NeiSSll, Martin W James, Stephen D Ryder, Guruprasad P Aithal

**Affiliations:** 1 NIHR Nottingham Biomedical Research Centre, Nottingham University Hospitals NHS Trust and University of Nottingham; 2 Nottingham Digestive Diseases Centre, University of Nottingham; 3 Department of Radiology, Nottingham University Hospitals, NHS Trust, Nottingham, UK

**Keywords:** hepatocellular carcinoma, transarterial chemoembolization, healthcare costs, drug-eluting beads, objective response

## Abstract

**Background:**

A meta-analysis comparing drug-eluting beads transarterial chemoembolization (DEB-TACE) with conventional transarterial chemoembolization (cTACE) has recently been published. On balance, no significant differences were found in terms of objective response and overall survival. The impact on healthcare costs had been studied in small series based on a hypothetical model and was in favor of DEB-TACE. We aimed to evaluate and compare health-care costs and effectiveness of both modalities in a cohort of patients from Nottingham, UK.

**Methods:**

Using a dedicated radiology database, we identified all patients who had undergone cTACE or DEB-TACE between 2006 and 2012 at a single tertiary referral center based in Nottingham. We collected clinical data, including treatment response, postprocedure complications and 30-day mortality. Costing models were constructed to present both our local hospital perspective as well as the national health service position.

**Results:**

During our study period, 101 procedures were performed on 43 patients (76 cTACE procedures on 26 patients and 25 DEB-TACE procedures on 17 patients). Overall, 11/26 in cTACE and 5/17 in DEB-TACE group had progressive disease (*p*=0.52). Adverse events were seen in 6/76 cTACE compared with 7/25 DEB-TACE group (*p*=0.16). Based on the predetermined standard pathway there was an unadjusted average cost difference of £3770.30 (TACE =£9070.44, DEB-TACE =£5300.14) in favor of the DEB-TACE. Results from our costing models indicated a £2715.33 (95% CI £580.88–4849.77) cost difference in favor of the same procedure.

**Conclusions:**

Even when the extra costs of DEB-TACE were considered, the overall treatment costs per patient were lower in relation to cTACE.

## Introduction

Hepatocellular carcinoma (HCC) is the most common primary malignancy of the liver and the second leading cause of all cancer-related mortality globally.^1^,^2^ The majority of cases are diagnosed late at advanced stages and deemed unsuitable for curative treatments.^3^ Transarterial chemoembolization (TACE) is commonly used for the management of unresectable primary liver cancer.^4^ It works by blocking the tumor feeding arterial supply using an embolizing agent and enabling a local injection of chemotherapy directly into the tumor. Multiple TACE sessions may be required to achieve a desirable outcome.

Response to TACE is commonly judged according to time-to-event, for example, progression as well as overall survival.^4^ The Modified Response Evaluation Criteria in Solid Tumors (mRECIST) is commonly used to objectively measure tumor response or progression. It is based on a radiological assessment of tumor size specifically relating to contrast enhancement.^5^ Sustainable objective response is achieved in ~35% of patients.^6^ The benefits of this treatment on 2-year survival have been established by meta-analysis of randomized controlled trials (RCTs) (odds ratio, 0.54; 95% CI: 0.33, 0.89; *p*=0.015).^7^

TACE with drug-eluting beads (DEB-TACE) as opposed to conventional TACE (cTACE) involves the injection of beads into hepatic artery branches offering simultaneous embolization with sustained and controlled drug release. Consequently, DEB-TACE offers a favorable pharmacokinetic profile and lower peak plasma concentrations of chemotherapy.^8^ DEB-TACE is associated with 50% 4-year survival based on a retrospective and non-comparative study.^9^

Five meta-analyses comparing the safety and efficacy of DEB-TACE in comparison with cTACE have been published within the last 2 years.^10^–^14^ The aforementioned studies had different selection criteria and methodologies and therefore report varying comparative outcomes on the safety and efficacy of both procedures. Cost comparisons between cTACE and DEB-TACE were performed in 2 studies. Vadot et al retrospectively analyzed healthcare costs within the French infrastructure and found a significantly shorter mean hospital admission duration for patients who received DEB-TACE. This led to a subsequently better reported economic profile in France.^15^ The results of the study were limited to France and further studies were required to confirm generalizability to other national healthcare systems. Cucchetti et al constructed a hypothetical cost effectiveness Markov simulation model on published studies investigating a total number of 1860 patients and found a lower incremental cost of DEB-TACE.^16^ We have evaluated the safety, efficacy and costs of DEB-TACE versus cTACE from real-life patients from a large UK-based tertiary care institution. We aimed to first, validate the results published from France and review their generalizability. Second, validate the results published on a large hypothetical cohort using Markov simulation. With the widespread economic concerns, this may have a quick translation impact on healthcare systems.

## Methods

### Ethics and consent statement

The study is registered by the Digestive Diseases and Thoracic Directorate of the Nottingham University Hospitals NHS Trust as an audit. Ethical approval and patient consent were not required for this retrospective study which was performed as a part of our service evaluation.

### Patient selection

We performed a retrospective analysis of patients who had cTACE or DEB-TACE between January 2006 and December 2012 for the treatment of HCC in a tertiary referral center. We identified the patients using a dedicated radiology database that is used to store and report procedural details as part of routine clinical practice. Clinical notes as well as electronic record information were used for data collection. We collected: 1) patient variables, including demographics, etiology of liver disease, Child–Pugh score and UK model for end-stage liver disease; 2) tumor variables, including size, location and number; 3) procedure variables, including number of TACE sessions required, length of hospital stay, postprocedure complications and 30-day mortality. Patients were excluded if they: 1) had not had follow-up in our unit; 2) had TACE for indications other than HCC; 3) had both cTACE and DEB-TACE on different occasions. Patients were followed up until discharge from clinic, including death or until the date of the last available cross-sectional imaging, if still under follow-up. Computerized tomography scans pre- and postprocedure were used to objectively assess tumor response using mRECIST. The images were reviewed by an expert radiologist as a part of the study. Cumulative tumor size of the 2 largest measurable lesions per liver was used where there was more than 1 lesion.^5^

### Statistical analysis

Differences between groups of continuous variables were assessed by unpaired *t*-tests (parametric data) or Mann–Whitney U (non-parametric data) tests. A *p*-value <0.05 was considered statistically significant. Possible predictors of objective response were fitted into a proportional ordered logistic regression model. This analysis was performed on R statistical software using R packages mass and ggplot2 (R core team, Vienna, Austria). TACE procedures were counted twice for the same patient if the interval between both procedures was more than 4 months and both were assessed by postprocedure cross-sectional imaging independently. Covariates were limited by data availability and included the following:

1. Interval in weeks from TACE to cross-sectional imaging
2. Tumor size at baseline
3. Vascular invasion and/or extra-hepatic disease, including porta-hepatis lymph nodes.

### Costs

In order to obtain per patient cumulative cost figures, a reference treatment pathway was constructed with the assumption that it would be followed by all patients during the study’s time. Follow-up procedures were modeled as shortened versions of the first procedure. Two different costing models were constructed in order to present both the local and the national perspectives. The local cost of the beads was added to both models since the national equivalent could not be identified. Details on the price weights can be found in Table 1. National Health Service (NHS) schedule of reference cost 2012–2013 was used to obtain the cost data.

### Cost analysis

To address the non-normality of distributions, costs were analyzed using generalized linear models (GLM) as described by Dunn et al.^17^ Regression on raw costs using ordinary least squares (OLS) was used for comparison. Covariates were chosen from the available dataset with the aim of maximizing the model’s explanatory power. To objectively measure clinical effectiveness, patient age, tumor size at presentation and last known mRECIST score were added to the model along with total costs and the treatment variable. Lowest Akaike information criterion and the modified Park test as described by Manning and Mullahy^18^ were used to evaluate the model’s goodness of fit. Three patients were excluded from the cost analysis due to lack of availability of sufficient data. Re-admissions were accounted for in our cost analysis.

## Results

### Patient outcomes

**Figure 1:**
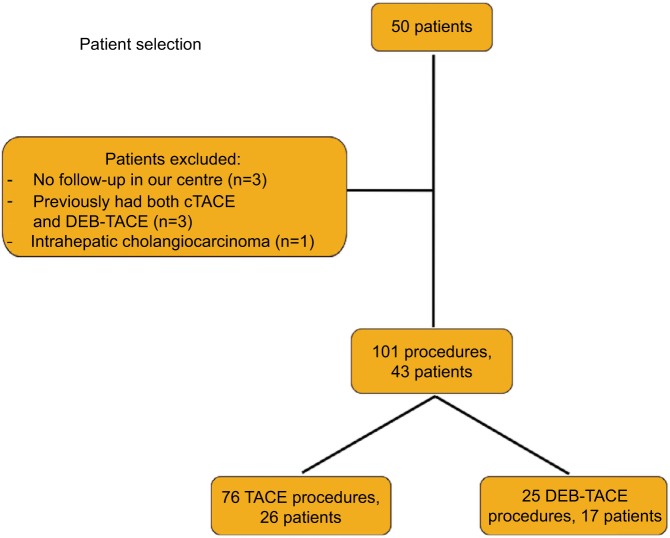
Summary of included and excluded patients and number of TACE procedures. **Abbreviations:** cTACE, conventional transarterial chemoembolization; DEB-TACE, drug-eluting beads transarterial chemoembolization; TACE, transarterial chemoembolization.

Fifty patients in total were identified. After exclusions, we analyzed 101 procedures (cTACE=76, DEB-TACE=25) performed on 43 patients (cTACE=26, DEB-TACE=17) as described in Figure 1. Descriptive statistics are outlined in Table 2.

The majority of patients were male in both groups, with similar ages. Included patients had variable etiologies as outlined in Table 2. Both groups were followed up for more than 1 year on average. Patients in the DEB-TACE group had a significantly smaller number of nodules within their livers (*p*=0.04). Despite this, the cumulative tumor size of all nodules per liver added together was not significantly different between both groups. DEB-TACE group required significantly less sessions (median=1) in comparison with cTACE (median=2.5) per patient (*p*<0.005).

### Mortality and complications

One patient in the DEB-TACE group died from biliary sepsis on day 14 of the same admission. In the cTACE group, there was a single 30-day mortality from hepatic decompensation; on day 2, postprocedure, the patient was transferred to a specialist palliative care facility. Severe transaminitis (>20× the normal) and hospital-acquired pneumonia occurred postprocedure in 1 patient in the cTACE group which prolonged admission in hospital by 5 days. One of the DEB-TACE cases had re-admission 6 days after the discharge from hospital with left hepatic artery dissection complicated by hospital-acquired pneumonia. The second admission had a duration of 7 days. There were no other notable severe complications otherwise, apart from limited transaminitis and postembolization syndrome (e.g., fever, nausea, vomiting or pain).

### Objective response

**Figure 2:**
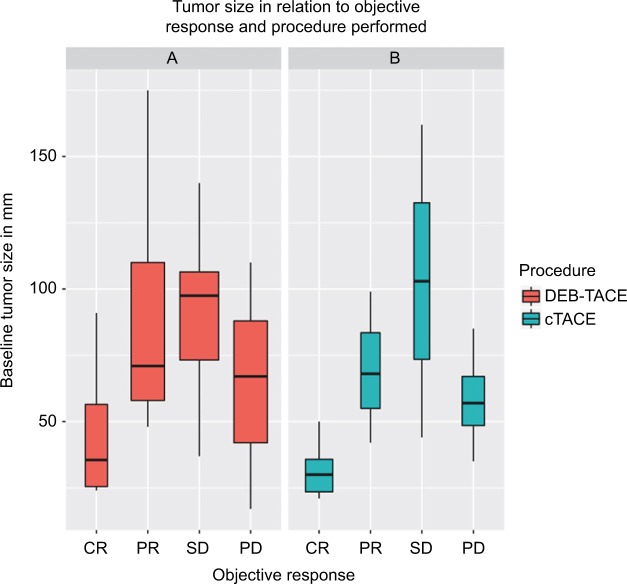
The target lesions treated by DEB-TACE (**A** - red) and cTACE (**B** - blue) are demonstrated. **Notes:** The figure shows objective responses in relation to tumor size as achieved by both modalities of TACE. Both modalities showed worse objective responses as the tumor size got bigger. PD outcome clearly did not follow the same trend. The width of each bar is proportional to sample size. **Abbreviations:** CR, complete response; cTACE, conventional transarterial chemoembolization; DEB-TACE, drug-eluting beads transarterial chemoembolization; PD, progressive disease; PR, partial response; SD, stable disease.

The method of TACE delivery (cTACE vs DEB-TACE) was not found to be independent predictor of objective response. Tumor size predicted objective response independent of all other variables as demonstrated in Figure 2 (*p*=0.0004, OR=1.035, 95% CI 1.017–1.057).

### Costs

The analysis of the cost data took into account 40 out of the 43 patients for whom there was a complete dataset. The cTACE and DEB-TACE arms differed in the added cost of the beads for the DEB-TACE and the average number of follow-up procedures per patient. Using the aforementioned pathway, the 2 procedures had an unadjusted average cost difference of £3770.30 (cTACE=£9070.44, DEB-TACE=£5300.14) based on the NHS costing and of £3253.68 (cTACE=£9033.19, DEB-TACE=£5222.89) based on local costing, in favor of the DEB-TACE arm. Results are summarized in Table S1. Upon comparing the output of both models, there was no significant benefit from the use of GLMs while the Park test did not highlight a specific model as the best fit. On the OLS regression model, the cost difference was at £2715.33 based on the national NHS costing and at £2746.58 based on our local costing. Treatment type was found to predict total costs independent of all other included covariates (*p*=0.014).

## Discussion and conclusions

We have demonstrated that overall treatment costs of DEB-TACE are lower than that of cTACE for the treatment of HCC. Our analysis is based on a real-life scenario. In the UK, all patients with HCC are managed through a regional cancer network with treatment decisions made by multidisciplinary teams (MDTs) based on predetermined pathways. Our economic model was based on the predetermined standard pathway which included the patient’s journey from time of referral to the tertiary center until the time of discharge, death or last follow-up. It included the costs of outpatient appointments, MDT discussion, inpatient stay, blood tests, TACE procedure and cross-sectional imaging. This economic modeling has shown an unadjusted average cost benefit of £3770.30 for patients receiving DEB-TACE compared with those who received cTACE. Patients undergoing DEB-TACE have required less number of procedures over the observed study period. The data suggest that contribution to the cost comparison is in favor of DEB-TACE with potential benefits to healthcare costs. Although neither the OLS regression nor any GLM had a significant explanatory power, there is an indication that the DEB-TACE arm can be less costly in the medium term. This is consistent with recently published data by French and Italian groups.^15^,^16^

Our limitations include the retrospective study design and possible selection bias, including the fact that patients treated by DEB-TACE had significantly fewer target liver lesions. The cost of beads reported in our study may lack generalizability as our analysis was based on our local purchase costs. However, the commissioning of health services is determined based on these estimates; so, these influence the design and delivery of services in the British NHS. The use of cTACE had been well established in our unit for many years. In contrast, DEB-TACE had been introduced as a relatively new technique. The choice of patients having DEB-TACE was mainly influenced by the time of introduction of the new service rather than specific clinical features. Similarly, classically, we will protocol cross-sectional imaging after 2 sessions of cTACE. However, as DEB-TACE was relatively new at the time of introduction, we protocoled cross-sectional imaging after each session of DEB-TACE in the majority of cases. Cross-sectional imaging after each session of cTACE may have led to different results. The study also highlights that, judging by objective response, we demonstrated that DEB-TACE is similar to cTACE. It also affirms the likelihood that its medico-economic consequences are more favorable in the medium term in comparison with cTACE.

There is some controversy in the literature, however, about the effect of DEB-TACE on objective tumor shrinkage.^19^ We therefore fitted our data into a logistic regression model and confirmed lack of direct effect from DEB-TACE on improved objective tumor response within our cohort. We found tumor size to be an independent predictor of objective response to TACE as would be expected. Interestingly, progressive disease (PD) did not seem to be limited by tumor size, that is, patients who had PD did not necessarily have larger tumors (Figure 2). This highlights the seemingly unstoppable role of tumor biology in dictating behavior and prognosis.

Based on the recent meta-analyses, DEB-TACE has no statistically significant impact on survival or objectively evaluated treatment response according to RCTs. In contrast, the largest meta-analysis to date that included non-RCT prospective and retrospective studies, demonstrated that DEB-TACE was associated with survival benefit.^13^ Another large meta-analysis including 4 RCTs and 6 retrospective studies found significant differences in the incidence of post-embolization syndrome or liver dysfunction (Table 3).^14^ Meta-analysis limited to RCTs found DEB-TACE to be safer, specifically in terms of myelosuppression and alopecia.^11^ Xie et al, reported significant improvement in objective response in the DEB-TACE group. The meta-analysis was subsequently heavily criticized by Kodama et al who reported no difference in objective response upon reproducing the results.^12^,^19^

It is worth noting that there are several limitations of reviewing studies based on meta-analysis. First, the number of TACE sessions required and practically performed was not compared. The studies did not report if less sessions were required by DEB-TACE to achieve the same outcome. Second, the size of beads and dosage of chemotherapy was variable. Third, the timing and criteria used for radiological assessments were heterogeneous. Finally, the patient populations, for example, model for end-stage liver disease score and stage of fibrosis was not unified. Better efficacy and safety remain a theoretical possibility based on the aforementioned results.

We conclude that DEB-TACE, a newer technique for the treatment of unresectable HCC, is an effective modality and our cost analysis shows it to have cost benefits when compared with cTACE. Prospective head-to-head trials on selected patient populations are needed to evaluate its efficacy as well as cost benefits to the healthcare system.

## Data sharing statement

The dataset is available from the corresponding author at (Guru.Aithal@nuh.nhs.uk). Consent was not obtained from participants but the presented data are anonymized and risk of identification is low.

## Supplementary material

**Table S1:** Cost models: (goodness of fit and ordinary least squares [robust])

Regression models	Incremental effect on cost	95% CI max	95% CI min	AIC
**Local costings**				
OLS (robust)	−£2,746.58	−£4,899.02	−£594.14	19.39
GLM (Gaussian-Log)	−£3,291.65	−£4,952.08	−£2,104.93	19.36
GLM (Gamma-ID)	−£2,589.00	−£4,676.21	−£501.80	20.19
GLM (Gamma-Log)	−£2,824.93	−£5,266.25	−£2,153.15	20.20
GLM (Inverse Gaussian-ID)	−£2,671.19	−£4,531.63	−£810.75	28.69
GLM (Inverse Gaussian-Log)	−£3,196.00	−£20,881.79	−£2,109.59	28.69
GLM (Poisson-ID)	−£2,609.35	−£2,667.73	−£2,550.97	1243.50
GLM (Poisson-Log)	−£2,970.33	−£4,794.18	−£2,083.05	1232.81
**National NHS costing**				
OLS (robust)	−£2,715.33	−£4,849.77	−£580.88	19.37
GLM (Gaussian-Log)	−£3,244.57	−£4,865.83	−£2,093.79	19.35
GLM (Gamma-ID)	−£2,557.90	−£4,635.49	−£480.31	20.21
GLM (Gamma-Log)	−£2,790.23	−£5,155.18	−£2,134.29	20.21
GLM (Inverse Gaussian-ID)	−£2,631.80	−£4,480.63	−£782.97	28.72
GLM (Inverse Gaussian-Log)	−£3,051.61	−£16,820.11	−£2,118.51	28.72
GLM (Poisson-ID)	−£2,580.16	−£2,638.81	−£2,521.51	1214.53
GLM (Poisson-Log)	−£2,933.00	−£4,712.09	−£2,069.84	1204.22

**Abbreviations:** AIC, Akaike information criterion; GLM, generalized linear model; NHS, National Health Service; OLS, ordinary least squares.

## Figures and Tables

**Table 1:** Price weights of TACE-related assessments and procedures from local and national perspectives

	Local price weights	National average price weights
Outpatient first appointment	£92	£225
Discussion at HPB MDT meeting	£110	£110
CT scan with contrast	£125	£106
Preoperative assessment	£76	£200
Hepatobiliary interventional radiology procedure	£2,596	£2,342
Outpatient follow-up appointment	£76	£200
Drug-eluting beads	£550	Not available

**Abbreviations:** CT, computerized tomography; HPB MDT, hepatobiliary multidisciplinary team; TACE, transarterial chemoembolization.

**Table 2:** Descriptive statistics of included patients in both groups

	DEB-TACE	cTACE	*p*-value
Number of patients	17	26	
Number of procedures	25	76	
Age, years (mean ± SEM)	67.76±3.05	68.35±2.42	0.88
Gender			
Male	14	24	
Female	3	2	
Etiology			
Unknown/noncirrhotic	5	10	
ALD	7	4	
HCV	2	3	
HBV	1	4	
NASH	2	3	
Other	0	2	
Number of tumors			
Multifocal	2	12	
Two lesions	1	6	0.04
Single lesion	14	8	
Size of tumor in mm (mean ± SEM)	55.35±7.44	75.85±7.25	0.065
Procedure per patient (mean ± SEM)	1.43±0.16	3.03±0.33	0.0007
Follow-up in months	12.8	17.6	0.13
Disease response (%) mRECIST			
SD (%)	10	20	
PD (%)	30	40	
PR (%)	15	30	
CR (%)	45	10	
Inpatient stay, days (mean ± SEM)	2.81±0.5	2.37±0.11	0.21
Complications	7/25	6/76	0.16
30-day mortality	1	1	

**Abbreviations:** ALD, alcohol related liver disease; CR, complete response; cTACE, conventional transarterial chemoembolization; DEB-TACE, drug-eluting beads transarterial chemoembolization; HBV, hepatitis B virus; HCV, hepatitis C virus; mRECIST, Modified Response Evaluation Criteria In Solid Tumors; NASH, nonalcoholic steatohepatitis; PD, progressive disease; PR, partial response; SD, stable disease; SEM, standard error of mean.

**Table 3:** Recently published meta-analysis comparing DEB-TACE to cTACE

Citation	Total studies	Study design	Patients	Surv	OR	AE
Zhou et al (2014)^10^	9	5 RCTs 3 retrospective	830	Better	Better	Same
Xie et al (2015)^12^	6	4 RCTs 0 retrospective	652	Same	Better	Same
Hui et al (2015)^11^	4	4 RCTs	527	NA	Same	Better
Facciorusso et al (2016)^14^	12	4 RCTs 6 retrospective	1449	Same	Same	Same
Chen et al (2017)^13^	16	4 RCTs 9 retrospective	1832	Better	Same	Same

**Abbreviations:** AE adverse events; cTACE, conventional transarterial chemoembolization; DEB-TACE, drug-eluting beads transarterial chemoembolization; NA, not available; OR, objective response; RCTs, randomized controlled trials; Surv, survival.
